# Simulation-based learning: Just like the real thing

**DOI:** 10.4103/0974-2700.70743

**Published:** 2010

**Authors:** Fatimah Lateef

**Affiliations:** Senior Consultant, Director of Training and Education, Department of Emergency Medicine, Singapore General Hospital, Singapore

**Keywords:** Simulation, learning, virtual reality, medical education

## Abstract

Simulation is a technique for practice and learning that can be applied to many different disciplines and trainees. It is a technique (not a technology) to replace and amplify real experiences with guided ones, often “immersive” in nature, that evoke or replicate substantial aspects of the real world in a fully interactive fashion. Simulation-based learning can be the way to develop health professionals’ knowledge, skills, and attitudes, whilst protecting patients from unnecessary risks. Simulation-based medical education can be a platform which provides a valuable tool in learning to mitigate ethical tensions and resolve practical dilemmas. Simulation-based training techniques, tools, and strategies can be applied in designing structured learning experiences, as well as be used as a measurement tool linked to targeted teamwork competencies and learning objectives. It has been widely applied in fields such aviation and the military. In medicine, simulation offers good scope for training of interdisciplinary medical teams. The realistic scenarios and equipment allows for retraining and practice till one can master the procedure or skill. An increasing number of health care institutions and medical schools are now turning to simulation-based learning. Teamwork training conducted in the simulated environment may offer an additive benefit to the traditional didactic instruction, enhance performance, and possibly also help reduce errors.

## INTRODUCTION

In medical education, there should be exposure to live patients so that medical students and doctors can acquire the necessary skills. There is also, on the other hand, an obligation to provide optimal treatment and to ensure patients’ safety and well-being. These two competing needs can sometimes pose a dilemma in medical education. Also, medicine is a discipline that is a science as well as an art and repeated exposures with enhanced experience will help improve skills and confidence.[1]

The growing complexities of patient care require doctors to master not only knowledge and procedural skills but also the ability to effectively communicate with patients, relatives, and other health care providers and also to coordinate a variety of patient care activities. Doctors have to be good team players and their training programmes must systematically inculcate these skills. Teamwork-related competencies are relatively new considerations in the arena of health care

## SIMULATION-BASED LEARNING

Simulation is a technique for practice and learning that can be applied to many different disciplines and types of trainees. It is a technique (not a technology) to replace and amplify real experiences with guided ones, often “immersive” in nature, that evoke or replicate substantial aspects of the real world in a fully interactive fashion. “Immersive” here implies that participants are immersed in a task or setting as if it were the real world.[2,3]

Full-body mannequin simulators originated in the field of anesthesia in the late 1960s, based on work done by Denson and Abrahamson from the University of Southern California. This model was known as ‘Sim One’ and was used for training in endotracheal intubation and induction of anesthesia. In the 1980s, during the time when personal computers became less expensive and more simulation software became available, independent groups began to develop simulator systems. Much of this was utilized in the areas of aviation, military training, nuclear power generation, and space flights. In the early 1990s, more comprehensive anesthesia simulation environments were produced, which included the MedSim and, later, the Medical Education Technologies Inc. (METI) Advanced Human Patient Simulator. Aviation simulation training concepts then begun to be gradually introduced into anesthesia and other areas of medicine like critical care, obstetrics, emergency medicine, and internal medicine. Current full-body simulator models incorporate computerized models that closely approximate the physiology seen in the human body.

Simulation-based learning can be the answer to developing health professionals’ knowledge, skills, and attitudes, whilst protecting patients from unnecessary risks. Simulation-based medical education can be a platform for learning to mitigate ethical tensions and resolve practical dilemmas. Simulationbased training techniques, tools, and strategies can be applied in designing structured learning experiences, as well as be used as a measurement tool linked to targeted teamwork competencies and learning objectives. Simulation-based learning itself is not new. It has been applied widely in the aviation industry (also known as CRM or crew resource management), anesthesiology, as well as in the military. It helps to mitigate errors and maintain a culture of safety, especially in these industries where there is zero tolerance for any deviation from set standards.[1,3]

Simulation has also begun to change much of the ways in which medicine is taught and how trainees and junior doctors acquire the relevant skills. Medical, nursing, and other health care staff also have the opportunity to develop and refine their skills, repeatedly if necessary, using simulation technology without putting patients at risk.[4] Simulation training centers, with their new techniques and equipment, offer unique opportunities for dynamic, complex, and unanticipated medical situations to be practiced and managed. In both aviation and health care domains, human performance is strongly influenced by the situational context, i.e., the interaction between the task, the environment, and the behavior of team members. In aviation, more than 50 years of research has shown that superior cognitive and technical skills are not enough to ensure safety: effective teamwork skill is a must. Similar observations are also now being made in the practice of medicine.[3–8]

The cost of simulation training, when it was first introduced, was high, and few institutions had the vision to realize that it was a worthwhile investment for the long term. It has indeed turned out to be a very flexible and durable form of medical education and training. Much of the cost is contributed to by the manpower or technician costs as well as cost of the laboratory setup and maintenance. The computer- and information technologycontrolled equipment advances medical learning and ensures that students and doctors learn procedures and treatment protocols before performing them on actual patients. The simulated environment allows learning and re-learning as often as required to correct mistakes, allowing the trainee to perfect steps and fine-tune skills to optimize clinical outcomes.[5,6] There can also be simulated examples or scenarios of rare or unusual cases that are often hard to come by in the clinical settings. The simulated situation and scenarios can give students and inexperienced junior doctors realistic exposure to such cases. It can certainly help in making books and lecture materials come alive. It helps ensure that students and trainees gain clinical experience without having to depend on chance encounters of certain cases. Many also believe that simulation-based learning enhances efficiency of the learning process in a controlled and safe environment.[9,10]

In the earlier days of medicine some form of “simulation” was already being applied in the form of case scenarios and the use of case presentations. These are also being utilized to assess candidates in the objective structured clinical examination (OSCE). Life support courses such as basic and advanced cardiac life support (BCLS and ACLS, respectively), as well as basic and advanced trauma life support (BTLS and ATLS, respectively), also utilize simulation techniques and principles for learning and testing.[5] Simulation is a tool for learning and training as well as for assessment of performance.[10,11]

The skills requirement which can be enhanced with the use of simulation include:

- Technical and functional expertise training
- Problem-solving and decision-making skills
- Interpersonal and communications skills or team-based competencies

All of these share a common thread in that they require active listening and collaboration besides possession of the basic knowledge and skills. With every training programme it is best to have feedback and debriefing sessions that follow. Feedback must be linked to learning outcomes and there must be effective debriefing protocols following all simulation exercises. Studies have shown that simulation improves learning.[5,6,9,11] Simulation is effective in developing skills in procedures that require eye–hand coordination and in those that call for ambidextrous maneuvers, such as bronchoscopy and other endoscopic procedures[9–11] Simulation training helps learners prepare to deal with unanticipated medical events, thus increasing their confidence.

Multidisciplinary teams deliver a multitude of health care services today but many organizations still remain focused on individual technical responsibilities, leaving practitioners inadequately prepared to enter complex team-based settings. When health care providers of different disciplines train separately, it may be difficult to integrate their capabilities. Effective multidisciplinary teams must always have good communications and leadershipsharing behavior, which can help ensure patient safety.

Inculcation of teamwork values is an example of the nontechnical, but essential, part of training of medical professionals. Simulation has the potential to create lasting and sustainable behavior and culture change that will make health care more effective and safer. It also has the ability to fundamentally alter learners’ ways of doing things and working with others. Transformational change can only come about when the learner recognizes the problems and then adopts a proactive approach to work on it and correct it.

The essence of a team is the shared goal and commitment. It represents a powerful unit of collective performance, which can be done as an individual or mutually. These must eventually translate common purpose into specific performance goals. One of the important ingredients of teams with good outcomes is the basic discipline of the team. Simulation training and practice affords the essentials for creating an effective medical team with a sense of group identity, group efficacy, and trust amongst members. There needs to be true engagement and understanding for team members to work together well. Examples of these can be seen in the incredible teamwork and excellent team dynamics that can exist during good resuscitation, certain surgery, and the more complex intensive care cases. Members who have had sufficient training and knowledge can be flexible enough to adapt to any new situation and break out of their ingrained routines and they get more proficient with time. Each member of such a health care team can carry out another team member’s job, which reflects their interdependence. A learning team will have some degree of substitution, defined roles and responsibilities, flexibility, good process flow, and an awareness of common goals. Conflict resolution is another aspect of teamwork that can be practiced during simulations.

Sexton *et al*. used a cross-sectional survey to assess errors, stress, and teamwork in medicine and the aviation industry. Medical staff reported that error is an important issue but difficult to discuss and that it was not being handled well in their hospital.[12] Other problems that were mentioned included different perceptions of teamwork amongst team members and reluctance of senior staff to accept inputs from junior members.[12]

## INTERDISCIPLINARY TEAMWORK AND SKILLS

The health care team comprises doctors from various disciplines, nurses, physiotherapists, radiologists and radiographers, pharmacists, medical students, and other personnel. The composition varies according to the objective of the teams; examples include stroke management teams, trauma teams, acute coronary syndrome intervention teams, etc. The training of each member of the team is decided by his or her own discipline. As such, there is a need to bring them together in an integrated fashion to learn how to manage a patient with complex medical problems. No one discipline is more important than the other. Everyone has a role to play. In simulated exercises involving teams, members learn how not “to step on each others’ toes.” They are made aware of their synergistic roles. There must also be some flexibility allowed at various junctures of decision-making and intervention. Team-work skills and interpersonal communication techniques are essential components of such training and exercise.[13–16]

The simulation trainers are often senior staff who have a good grasp and helicopter view of the whole team-based approach. They must be able to objectively view the group dynamics and interaction within the teams they train and provide valuable feedback. They will assess the team’s performance in real-time and may maintain checklists of activities, actions, and relevant human factors. Videotaping the role-playing is useful as it can be played back and the highlights shared with the team as part of their learning process. Trainers can points out both the negative and positive practices and behaviors to the participants.[16,17]

There are also scenario writers for these simulation cases. These writers can customize the scenarios for interdisciplinary team training and role-playing in order to highlight or facilitate certain roles or team interaction. These scenarios should be realistic, practical, and comprehensive. Scenarios would usually also have event triggers, environmental distractors, and supporting events. They should be developed systematically with proficiency-based assessment in place, which can emphasize integrative team performance as well as technical performance. All practice and action should also be validated by data and evidence.[16]

Some common pitfalls that have been observed during team performance include:[17,18]

- The lack of understanding of roles and responsibilities of other team members, particularly across disciplines.
- The absence of clearly defined specified roles may persist, despite generally acceptable team performance; this may not become obvious until there is a change in team members, which then reveals the role confusion.
- Most health care systems have no or few processes or backup plans when errors occur.
- There is an unspoken assumption by members that everyone will perform at 100% efficiency and effectiveness. However, there is no method to measure this.

## SETTING UP A SIMULATION TRAINING CENTER

A simulation center would be a long-term investment in medical education. It can be used for undergraduate training (such as in the study of anatomy, physiological functions, familiarization with medical examination techniques), for residency training (e.g., in refining and mastering procedural skills and techniques or preparing for practical examinations, refresher courses, and recertification tests etc.), for continuing medical or nursing education (e.g., training in practical skills), or for competency testing prior to recruitment.[11] To start off, there must be a convenient location, usually somewhere on the hospital or university campus for convenience of proximity. The architecture plan and infrastructure must be decided upon in consultation with the trainers/end-users of the center. It must include adequate space for training small groups, rooms with one-way mirrors, and sufficient space for equipment setup, amongst other facilities. There must also be provision for video recording equipment. Manpower would include full-time technicians and a manager; the trainers are usually part-time medical personnel. The decision to purchase suitable mannequins and equipment must only be made after adequate demonstration and trials have been done and all parties are satisfied. It is also important to have technical support from the vendors in the long term. The different forms of medical simulation technology training that can be considered for the center would include:

1. *Human patient simulators*: The centerpiece is usually a fullsized patient simulator that blinks, breathes, and has heart beat, pulse, and respiratory sounds. This mannequin can be very technologically advanced. For example, it may “interact” with learners through computer-guided teaching programmes Attached monitors can display vital signs and this can provide virtual simulation of almost every major bodily function. This simulator can be used for scenarios from simple physical examination to interdisciplinary major trauma management. Some simulators can even recognize injected medications via a laser bar-code reader and then respond with appropriate vital sign changes
2. *Simulated clinical environment*: An intensive care unit, emergency room cubicle, or operating room is prepared with all the equipment and the crash cart. The setup is as realistic as the actual facility. Trainees can familiarize themselves with the setup and arrangements.
3. *Virtual procedure stations*: Various stations can be set up, depending on what the focus is. These stations will have all the relevant equipment and setup for the procedure to be carried out, e.g., bronchoscopy, colonoscopy, intubation. The simulators can present a variety of different scenarios and pathologies and the trainee can practice until he/she masters the technique(s).
4. *Electronic medical records*: As more health care institutions adopt electronic medical records to track and to manage patients, this can also be a station setup in the center. The system utilized will have fictitious patients with their histories, notes, and lab results. There may also be system integration, such as the link between records and the laboratory as well as the radiology results (digitalized radiographs).

Currently, adult simulation equipment and mannequins are already well established. Pediatric ones are still in the experimental stage, but there will be future developments. There are children’s hospital which are already using simulation-based training for their staff.

For institutions that cannot afford to set up an entire simulation laboratory, a less expensive option could be to invest in simulation mannequins only. This could be purchased in different numbers and be used for training purposes. Institutions and their leaders must learn to accept the candidates with an open mind. The leaders must be strict with their education and training portions. It may also be useful to plan visits to established simulation centers.

## SAFETY AND SIMULATION

Health care safety can be compared to other high-stakes industries such as aviation, the military, and nuclear power generation. In these industries, safety depends on the prevention of human errors and on engineered redundancy so that the systems work without failing. Morbidity and mortality can be the consequences of failures in these environments.[20–23] Hospitals do include the numbers of medical errors as one of their key performance indicators. The utility of simulation in health care is certainly most interesting to consider in the context of patient safety.[23–25]

One important concept in medical safety is the paradigm of how one learns. Traditionally, medicine works on the apprenticeship model. Trainees and residents begin caring for patients on their first day of internship under the supervision of more experienced staff, who provide a safety net for errors. Despite their learning about medical care before assuming responsibility of their first patient, there must indeed be a first time for the performance of high-risk procedures, resuscitation, and the implementation of critical decision-making skills in real time on real patients. Simulation provides a learning model to complement traditional learning in medicine. These scheduled simulation exposures can ensure the residents have exposure to these emergencies, even if they are only simulated scenarios. For the performance of procedures, it has been shown that the volume of experience decreases patient complication rates. Simulators do allow for the development of experience prior to performance of these procedures on patients.[4,11,23–25]

The apprenticeship model of medical teaching has not been widely studied, but newer methods which are resourceful and intensive have been given greater scrutiny. The body of literature is gradually emerging. A general scan of the literature from 1969 to 2003 concluded that the rigor and quality of research in simulation needs improvement, although high-fidelity simulations are educationally effective and complement traditional teaching in patient care settings. The features of simulation which best facilitate learning include:[23,26]

- The ability to provide feedback
- Repetitive practice
- Curriculum integration
- The ability to range the difficulty levels

The educational benefits of simulation in medical education include the following:

- Deliberate practice with feedback
- Exposure to uncommon events
- Reproducibility
- Opportunity for assessment of learners
- The absence of risks to patients

To date, however, there have been no studies to show that simulation training improves patient care outcomes directly. There may be some reasons for this. Life-threatening complications are rare. Most institutions have quality improvement measures in place and selecting for the impact of simulation on patient outcomes can be difficult. However, there exist a significant body of data and evidence for the benefit of simulation training in educational outcomes. Learners who go through simulation do perform better on subsequent simulated tests and tasks. In a cohort study on medical students from five institutions, one group was exposed to 2 weeks of deliberate practice of cardiac bedside skills using the Harvey Cardiology Patient Simulator followed by 2 weeks of traditional ward work, while the other group went through 4 weeks of traditional ward training. The simulation group performed at twice as well as the ward group, with only half the training time.[24]

Devita *et al*. showed that simulated patients had better outcomes if doctors were trained to work together by reliably performing preassigned roles during a simulator exercise.[25]

## FUTURE DIRECTIONS

Simulation appears to be here to stay. Perhaps there will be a day when we may use it as a tool in evaluating candidates for medical school admission, just as dental students are put through some manual dexterity tests. As medical simulation games are being developed, medical training may change to include a portion of time dedicated to learning through gaming. More studies and research are also needed to determine whether simulation improves patient outcomes. Designers will continue to improve the technology of virtual reality to make experiences as seamless as possible.[26]

## CONCLUSION

Simulation-based training has opened up a new educational application in medicine. Evidence-based practices can be put into action by means of protocols and algorithms, which can then be practiced via simulation scenarios. The key to success in simulation training is integrating it into traditional education programmes. The clinical faculty must be engaged early in the process of development of a programme such as this. Champions and early adopters will see the potential in virtual reality learning and will invest time and energy in helping to create a curriculum. They can then help to engage the wider medical community. Teamwork training conducted in the simulated environment may also offer an additive benefit to the traditional didactic instruction, enhance performance, and possibly also reduce errors. The cost-effectiveness of potentially expensive simulation-based medical education and training should be examined in terms of improvement of clinical competence and its impact on patient safety. Perhaps, with the adoption of simulation as a standard of training and certification, health care systems will be viewed as more accountable and ethical by the population they serve.
